# Consensus on diagnosis and management of non-metastatic castration resistant prostate cancer in Brazil: focus on patient, selection, treatment efficacy, side effects and physician's perception according to patient comorbidities

**DOI:** 10.1590/S1677-5538.IBJU.2020.0249

**Published:** 2021-02-03

**Authors:** Fernando Maluf, Andrey Soares, Guilherme Avanço, Aline Lury Hada, Ana Paula Garcia Cardoso, Arie Carneiro, Daniel Herchenhorn, Denis Leonardo Fontes Jardim, Fabio Augusto Schutz, Fabio Roberto Kater, Felipe Moraes Toledo Pereira, Fernando Sabino Marques Monteiro, Igor Alexandre Protzner Morbeck, João Francisco Navarro Reolon, Karine Martins da Trindade, Livia Maria Querino da Silvo Andrade, Lucas Mendes Nogueira, Renato Furoni, Ricardo Azze Natel, Rodolfo Borges dos Reis, Rodrigo Nogueira Fogace, Vinicius Carrera Souza

**Affiliations:** 1 Hospital Beneficência Portuguesa Departamento de Oncologia São Paulo SP Brasil Departamento de Oncologia, Hospital Beneficência Portuguesa de São Paulo, SP, Brasil; 2 Hospital Israelita Albert Einstein Departamento de Urologia São Paulo SP Brasil Departamento de Urologia, Hospital Israelita Albert Einstein, São Paulo, SP, Brasil; 3 Centro Paulista de Oncologia São Paulo SP Brasil Centro Paulista de Oncologia, São Paulo, SP, Brasil; 4 Americano de Oncologia Cooperativa Grupo Latino Porto Alegre RS Brasil Grupo Latino - Americano de Oncologia Cooperativa, Porto Alegre, RS, Brasil; 5 Hospital Sírio Libanês Sociedade Beneficente de Senhoras São Paulo SP Brasil Sociedade Beneficente de Senhoras-Hospital Sírio Libanês, São Paulo, SP, Brasil; 6 Hospital Universitário de Brasília Unidade de Oncologia Brasília DF Brasil Unidade de Oncologia, Hospital Universitário de Brasília, Brasília, DF, Brasil; 7 Hospital Santa Lucia Centro de Oncologia e Hematologia Brasília DF Brasil Centro de Oncologia e Hematologia, Hospital Santa Lucia, Brasília, DF, Brasil; 8 Universidade Catolica de Brasilia Disciplina de Urologia Taguatinga DF Brasil Disciplina de Urologia, Universidade Catolica de Brasilia, Taguatinga, DF, Brasil; 9 Hospital Haroldo Juacaba Serviço de Urologia Fortaleza CE Brasil Serviço de Urologia, Hospital Haroldo Juacaba, Fortaleza, CE, Brasil; 10 Hospital São Rafael Serviço de Urologia Salvador BA Brasil Serviço de Urologia, Hospital São Rafael, Salvador, BA, Brasil; 11 Universidade Federal de Minas Gerais Hospital das Clínicas Departamento de Cirurgia Belo Horizonte Minas Gerais Brasil Divisão de Urologia e Departamento de Cirurgia, Hospital das Clínicas, Universidade Federal de Minas Gerais - Belo Horizonte, Minas Gerais, Brasil; 12 Universidade Cidade de São Paulo São Paulo SP Brasil Universidade Cidade de São Paulo, São Paulo, SP, Brasil; 13 Universidade de São Paulo Faculdade de Medicina da Universidade Ribeirão Preto SP Brasil Faculdade de Medicina da Universidade de São Paulo, Ribeirão Preto, SP, Brasil; 14 Clínica AMO Salvador Bahia Brasil Clínica AMO, Salvador, Bahia, Brasil

**Keywords:** Prostate cancer, familial [Supplementary Concept], Prostatic Neoplasms, Castration-Resistant, Consensus

## Abstract

**Background:**

Non-metastatic castration resistant prostate cancer (M0 CRPC) has seen important developments in drugs and diagnostic tools in the last two years. New hormonal agents have demonstrated improvement in metastasis free survival in M0 CRPC patients and have been approved by regulatory agencies in Brazil. Additionally, newer and more sensitive imaging tools are able to detect metastasis earlier than before, which will impact the percentage of patients staged as M0 CRPC. Based on the available international guidelines, a group of Brazilian urology and medical oncology experts developed and completed a survey on the diagnosis and treatment of M0 CRPC in Brazil. These results are reviewed and summarized and associated recommendations are provided.

**Objective:**

To present survey results on management of M0 CRPC in Brazil.

**Design, setting, and participants:**

A panel of six Brazilian prostate cancer experts determined 64 questions concerning the main areas of interest: 1) staging tools, 2) treatments, 3) side effects of systemic treatment/s, and 4) osteoclast-targeted therapy. A larger panel of 28 Brazilian prostate cancer experts answered these questions in order to create country-specific recommendations discussed in this manuscript.

**Outcome measurements and statistical analysis:**

The panel voted publicly but anonymously on the predefined questions. These answers are the panelists' opinions, not a literature review or meta-analysis. Therapies not yet approved in Brazil were excluded from answer options. Each question had five to seven relevant answers including two non-answers. Results were tabulated in real time.

**Conclusions:**

The results and recommendations presented can be used by Brazilian physicians to support the management of M0 CRPC patients. Individual clinical decision making should be supported by available data, however, for Brazil, guidelines for diagnosis and management of M0 CRPC patients have not been developed. This document will serve as a point of reference when confronting this disease stage.

## INTRODUCTION

Non-metastatic castration resistant prostate cancer (M0 CRPC) affects between 2-8% of prostate cancer (PCa) patients. M0 CRPC is defined when: 1) androgen deprivation therapy (ADT) leads to castration resistance, and 2) there are no metastases detectable according to conventional imaging (bone scan and negative computer tomography of chest, abdomen and pelvis). Additionally, according to the Prostate Cancer Working Group 2, patients must present with prostate-specific antigen (PSA) progression at an increase of 25% from nadir at a minimum rise of 2ng/mL and confirmed with a second value (1, 2). Therefore, M0 CRPC is a very specific diagnosis limited by a sensitive period of time.

Despite no detectable metastases through conventional imaging, nearly 60% of these patients develop metastatic disease during the first 5 years (3). Skeletal related events (SREs) associated with bone metastases, such as severe pain, pathological fractures, nerve and spinal cord compression, skull base involvement, need for radiation and/or surgery, lead to a significant reduction in health-related quality of life (QOL) and are associated with worse outcome. Of note, SREs have a negative impact on the health care system due to increased costs (4, 5).

M0 CRPC is a heterogeneous disease, for example, a prospective study included 331 patients reported that PSA level greater than 13.1ng/mL was associated with worse overall survival (OS) (relative risk, 2.34, 95% CI, 1.71 to 3.21, p <0.001) and bone metastasis-free survival (MFS) (relative risk, 1.98, 95% CI, 1.45 to 2.70, p <0.001). Additionally, PSA velocity was associated with worse outcomes (6). Until recently, there are limited number of diagnostic and staging tools and scarce treatment options with no clinical meaningful benefit for patients according to randomized trials (7–9).

In the last few years, new hormonal agents (NHAs) have been introduced in several PCa disease stages, including metastatic castration sensitive disease (M0 CSPC) and M0 CRPC. These NHAs have demonstrated improvement in MFS for M0 CRPC and have been approved by the U. S. Food and Drug Administration (FDA) and the Brazilian Health Regulatory Agency (ANVISA). Additionally, newer imaging tools have been introduced and are significantly increasing the detection of lymph-node and distant metastasis in PCa patients, mainly in patients with biochemical recurrence. However, these new imaging tools have not been included as part of the most recent M0 CRPC clinical trials (e.g., SPARTAN and PROSPER) (10, 11).

Currently, there are no treatment guidelines for M0 CRPC in Brazil. Given that new scientific developments are continuously happening, staying up to date with best practices can be challenging. As a result, a group of Brazilian experts reviewed the current management of M0 CRPC in the country through a consensus conference on staging, diagnosis, and treatment with a focus on efficacy and safety.

## METHODOLOGY

A small panel of six PCa physicians who specialize in genitourinary malignancies determined the five most relevant issues concerning the diagnosis and treatment of patients with M0 CRPC. These five topics included 1) staging tools, 2) treatments, 3) drug selection, 4) side effects of systemic treatment, and 5) use of osteoclast targeted therapy for SREs and symptomatic skeletal events (SSE) prevention. A total of sixty-four survey questions were developed on these five topics. Each question had five to seven relevant answers including two non-answers (“Abstain” and “Unqualified to Answer”). The two non-answers were provided for quality control, as a self-randomly selected answer could potentially skew results. The full panel for this consensus consisted of 28 multi-disciplinary cancer physicians from varying regions of Brazil with different realities regarding diagnostic and therapeutic resources. For all the questions, unless stated otherwise, it was assumed that for that specific recommendation, therapies (i.e. type of surgery, type of radiation therapy, drug) were approved and available. Therapies not yet approved in Brazil were excluded from the answer options. Each question was deemed “consensus” if 75% or more of the full panel had selected a particular answer. A review of the supporting literature as well as majority statements are included in each of the sections of this document and practical recommendations are provided.

## STAGING TOOLS FOR M0 CRPC PATIENTS

PSA levels and PSA doubling time (PSAdt) are important tools to classify prostate cancer risk. Conventional imaging technologies such as bone scan, abdomen and pelvic magnetic resonance imaging (MRI), positron emission tomography (PET) and computed tomography (CT) scans are important tools in staging PCa patients. Emerging imaging technologies for PCa have proved to be powerful adjuncts to conventional imaging for patients suspected of M0 CRPC and have a variety of approaches. The emerging imaging methods, such as 11C-choline PET/CT scans, 68Ga-labelled prostate-specific membrane antigen (PSMA), Sodium Flouride (NaF) PET scan, and whole-body MRI, are more sensitive to staging PCa as these methods detect metastasis earlier than conventional methods (12, 13). A recent retrospective study included 200 M0 CRPC patients diagnosed by conventional imaging at high risk of developing metastasis and assessed them with PSMA PET/CT. PCa lesions were detected in 196 (98%) of the patients, and 54% had metastatic disease demonstrating the improved sensitivity of PSMA PET/CT to conventional methods (13). Of note, all patients in this analysis had an absolute PSA level 3 2ng/mL and PSAdt <10 months, therefore, we can speculate if the results would be in the same for patients with lower absolute PSA levels or those with PSAdt ³ 10 months.

While PSA levels are clearly defined through testing, PSAdt can be more problematic to calculate, as there have been many methods suggested. However, PSAdt remains a vital tool for assessing a patient's prognosis especially for biochemical recurrence patients, both M0 CSPC and M0 CRPC. Alarmingly, PSAdt measures can vary significantly depending on the calculation method used. Many experts have called for the standardization of this formula in the clinical setting and in literature, as it can be the only parameter for treatment in settings that lack radiographic evidence (14). Regarding the preferred methodology for calculating PSAdt, two thirds of the panel (66%) stated they use a calculator and the last three PSA values in serum. The PCa Radiographic Assessments for Detection of Advanced Recurrence (RADAR III) Group's 2018 recommendations suggest follow-up images every 6-12 months, or more frequently based on a PSAdt of less than 6 months and/or symptoms in patients undergoing therapy for M0 CRPC (15).

The panel did not reach consensus in any of the questions related with staging tools and specifically the ones listing imaging methods. As a result, the panel was divided between the PET-CT with PSMA (or PET-MRI with PSMA) ± pelvic MRI (50% of the votes) and the CT of the Thorax or Chest X-Ray, CT of the abdomen and pelvis (or pelvic MRI) and Bone Scan (42% of the votes). These options are consistent with individual clinical practice choices and could be related with available technology that may vary significantly among different regions in the country and may create a bias according to where the voting physicians work. Of note, PET-CT with PSMA (or PET-MRI with PSMA) are not available in mostly public health and in some private practice centers. In this setting, the National Comprehensive Cancer Network (NCCN) guidelines version 1 2020 (17) supports the use of conventional image. Furthermore, the RADAR III group recommends a bone scan and a CT scan when the PSA reached 2ng/mL, and, if this is negative, the tests should be repeated when the PSA reaches 5ng/mL, and again after every doubling of the PSA every three months for asymptomatic men (15). It is currently unknown whether the improvement in the sensitivity and specificity of the next generation imaging studies will change the management of and outcome in M0 CRPC patients (13). Aside from systemic treatment, it is still unknown whether focal therapy for this patient population will have an impact on the outcome, however, other treatment alternatives can be used to try to slow progression if disease progression is detected early.

## TREATMENT FOR M0 CRPC PATIENTS

Until recently, treatment strategies for M0 CRPC patients included 1) observation and maintenance with ADT with serial imaging until metastatic findings or 2) other hormonal option supported by low evidence level trials (18). Three clinical trials (SPARTAN (10), PROSPER (11) and ARAMIS (18)) using MFS as the primary end point, showed that adding an NHA to ADT reduced the risk of developing metastatic CRPC or death compared to placebo. The SPARTAN trial examined apalutamide, which increased MFS time by 20.3 months when compared to placebo (median 40.5 vs. 16.2 months; HR=0.28, p <0.0001). The PROSPER trial, which examined enzalutamide, demonstrated superiority compared to placebo and included time to PSA progression, time to the first use of a subsequent antineoplastic therapy, and PSA response rate as secondary end points. However, median OS was not reached in either the SPARTAN or PROSPER clinical trials. Lastly, QOL assessments indicated substantial changes were the same for both groups (11). In the SPARTAN trial, other secondary end points included time to metastasis, progression-free survival, and time to symptomatic progression, which were all longer with apalutamide compared to placebo (10). Just before this consensus survey, the interim results from the ARAMIS trial had been posted. In this trial, darolutamide also reached the primary endpoint and significantly increased the MFS by 22 months (hazard ratio for metastasis or death in the darolutamide group, 0.41, 95% confidence interval, 0.34 to 0.50, p <0.001). However, since darolutamide had not approved by the FDA or ANVISA by the time of this consensus, darolutamide was not included as a treatment option for the survey.

To answer the questions on treatment of M0 CRPC in Brazil, the panel agreed to assume ideal scenario based on the best clinical evidence available. Each question proposed scenarios of men with various 1) life expectancies, 2) PSAdt quantities, and 3) PSA levels.

The votes of the panel showed consensus on the initial treatment for M0 CRPC patients with life expectancy greater than 10 years and PSAdt less than 10 months, regardless PSA levels. The panel reached consensus and would start the patient on apalutamide or enzalutamide in both cases (for PSA levels >2ng/mL 96% of the votes; for PSA levels <2ng/mL 84% of the votes). For patients with life expectancy lower than 10 years and a PSAdt less than 10 months, the panel was also in consensus (92%) to start apalutamide or enzalutamide when PSA was greater than 2ng/mL. These results are in accordance with the NCCN guidelines version 1.2020 (16), which support apalutamide or enzalutamide (both categories 1) regardless of the PSA level in patients with PSAdt less than 10 months mostly because this is the most robust factor for bone metastases in this setting (6).

Additionally, there was strong consensus for treatment of patients with life expectancy greater than 10 years and PSA greater than 2ng/mL but with a PSAdt of more than 10 months. In this case, 96.2% of the panel voted they would not start any specific systemic therapy and would consider observation as the best option. Absolute consensus (100%) was reached in the scenario of life expectancy greater than 10 years, PSAdt is longer than 10 months, and PSA is lower than 2ng/mL. In this case, the entire panel chose to not start any specific systemic therapy and considered observation was the best approach. Again, these results are in accordance with the NCCN guidelines version 1.2020 (16), which do not support the use of apalutamide or enzalutamide (both categories 1) in this setting. This guideline is due to the fact that longer PSAdt is a strong prognostic factor for delayed bone metastases and that there are patients who are metastasis free for a long period of time (6).

## DRUG SELECTION AND COMORBIDITIES

Standard of care treatment for M0 CRPC patients has been lacking. Variations range from observation using PSADT with serial imaging until metastasis being documented to hormonal agents despite lack of definite survival evidence for patients who showed shorter PSADT (17). New drug approvals of apalutamide, enzalutamide, and darolutamide are changing the landscape of the disease and increasing the treatment options for M0 CRPC patients.

Both apalutamide and enzalutamide have received approval from the FDA and ANVISA, however, darolutamide is currently only approved by the FDA. While most trials use OS as the primary endpoint observed, the efficacy and safety of these drugs were determined by MFS. Both the NCCN and the American Urological Association (AUA) suggest apalutamide, enzalutamide, or darolutamide as first line treatment options when PSAdt is equal to or less than 10 months (16, 19).

While PROSPER included QOL as a secondary outcome measure, SPARTAN and ARAMIS included QOL as an exploratory outcome measure. All trials used the Functional Assessment of Cancer Therapy––Prostate (FACT-P) Global Score and the European Quality of Life-5 Dimensions (EQ-5D-3L) questionnaires. PROSPER results showed that the median time to degradation was the same in both the enzalutamide and placebo groups. Both SPARTAN and ARAMIS results showed that the QOL of both groups (apalutamide/darotulamide and placebo) were generally the same over time.

The panel responded questions related to drug of choice for M0 CRPC patients with specific comorbidities and clinical scenarios with apalutamide, enzalutamide, or either. This exemplifies that physicians in the clinical setting are treating M0 CRPC patients with the most up to date evidence supported information. These results are unique due to the fact that no guidelines have fully explored this issue by selecting the agents in accordance to the toxicities (16, 19). For patients with a reasonable life expectancy and diabetes or mild/moderate hypertension, the panel reached consensus selecting “apalutamide or enzalutamide” as their choice of treatment (96% for diabetes, 88% for mild hypertension and 84% for moderate hypertension). Patients with severe hyper-tension and cardiovascular disease, either coronary disease or cardiac insufficiency greater than grade 2, would be treated with either “apalutamide or enzalutamide” by the majority of the panel, however, no consensus was reached that the panel would use one of these two drugs for the remaining clinical scenarios (71% for severe hypertension, 67% for cardiac insufficiency greater than grade 2 and 52% for coronary disease).

For M0 CRPC patients with a reasonable life expectancy and renal insufficiency or chronic obstructive pulmonary disease (COPD), the panel reached consensus that “apalutamide or enzalutamide” are their drugs of choice with 84% for renal insufficiency and 100% for COPD. The panel was divided when selecting the drug of choice for M0 CRPC for patients with past history of falls and a reasonable life expectancy between apalutamide (37%) and enzalutamide (33%) or either (30%).

The only questions where apalutamide was chosen over enzalutamide were regarding comorbidi-ties related to the central nervous system. As a result, the majority of the panel would choose apalutamide over any other drug if the M0 CRPC patient also had past history of seizure (78%), used medication that increased the risk of seizure (78%), had a mental impairment (81%), or psychiatric disorders (72%). However, theoretically, darolutamide which was not one of the listed treatment options, may have an even lower risk of seizure given it does not cross the blood-brain barrier.

Finally, the panel would check drug-drug interactions (75%) before making clinical decisions for M0 CRPC patients with multiple medications and a reasonable life expectancy. However, they did not reach consensus given some of the treating physicians would treat the patient with “apalutamide or enzalutamide” (14%) or only with apalutamide (11%).

## SIDE EFFECTS AND IMPLICATIONS OF MEDICATION SELECTION IN M0 CRPC

Based on the indications described, enzalutamide and apalutamide have had major quantitative impact in the management of M0 CRPC patients. However, if PSAdt is greater than 10 months, observation is still an option, given there are clinical drawbacks for both of these treatments. Of note, PSAdt greater than 10 months was not a part of the inclusion criteria in any of the clinical trials, including PROSPER, SPARTAN, and ARAMIS. Drawbacks for these alternatives include treatment toxicity and decreased QOL, which may be unnecessary burdens for a patient with a long-life expectancy. Potential adverse events (AEs) that decrease QOL for either of these drugs include fatigue, fractures, falls, hypertension, cardiovascular complications, hot flashes, nausea, loss of appetite mental-impairment disorders, seizures, rashes, among others.[Table t1]

**Table 1 t1:** Survey Questions and Answer Percentages from the Consensus on Diagnosis and Management of Non-Metastatic Castration Resistant Prostate Cancer in Brazil

BIOCHEMICAL RECURRENT CASTRATION-RESISTANT PROSTATE CANCER (M0 CRPC)								
1. STAGING TOOLS								
Which imaging method is indicated for a castration- resistant patient with biochemical recurrence after…		a) PET-CT with PSMA (or PET-MRI with PSMA) +/− pelvic MRI	b) Whole body MRI	c) Bone Scan	d) Pelvic MRI	e) Pelvic MRI and Bone Scan	f) CT of the Thorax or Chest-X-Ray, CT of the abdomen and pelvis (or pelvic MRI) and Bone Scan	g) None
1	Radical prostatectomy?	14 (50%)	––	––	––	2 (8.3%)	12 (41.7%)	––
2	Curatively intended radiotherapy?	15 (52%)	––	––	––	––	13 (48%)	––
3	What is your preferred methodology for calculating PSA doubling time (dT)?							
	a) I do not calculate PSA dT	b) Last two values (>0.1) using a manual tool	c) Last two values (>0.1) using an electronic calculator	d) Last three values (>0.1) using a manual tool	e) Last three values (>0.1) using an electronic calculator	f) At least four values (>0.1) using a manual tool	g) At least four values (>0.1) using an electronic calculator	
	––	––	1 (4.2%)	4 (12.5%)	18 (66.7%)	1 (4.2%)	4 (12.5%)	
2. TREATMENT								
What is your management of M0 CRPC in the majority of men with a life expectancy > 10-15 years and with…		a) Apalutamide or Enzalutamide	b) Apalutamide	c) Enzalutamide	d) Bicalutamide or Flutamide	e) Docetaxel	f) I would not start any specific systemic therapy until the development of clinical metastases	
4	PSADT ≤ 10 months and PSA ≥ 2ng/mL?	27 (96.2%)	1 (3.9%)	––	––	––	––	
5	PSADT ≤ 10 months and PSA < 2ng/mL?	24 (84%)	2 (8%)	––	1 (4%)	––	1 (4%)	
6	PSADT > 10 months and PSA ≥ 2ng/mL?	––	––	––	1 (3.9%)	––	27 (96.2%)	
7	PSADT > 10 months and PSA < 2ng/mL?	––	––	––	––	––	28 (100%)	
What is your management of M0 CRPC in the majority of men with a life expectancy < 10-15 years and with…		a) Apalutamide or Enzalutamide	b) Apalutamide	c) Enzalutamide	d) Bicalutamide or Flutamide	e) Docetaxel	f) I would not start any specific systemic therapy until the development of clinical metastases	
8	PSADT ≤ 10 months and PSA ≥ 2ng/mL?	26 (92.3%)	––	––	––	––	2 (7.7%)	
9	PSADT ≤ 10 months and PSA < 2ng/mL?	18 (65.4%)	1 (3.9%)	––	––	––	9 (30.8%)	
10	PSADT > 10 months and PSA ≥ 2ng/mL?	1 (3.9%)	––	––	1 (3.9%)	––	26 (92.3%)	
11	PSADT > 10 months and PSA < 2ng/mL?	––	––	––	––	––	28 (100%)	
12	In a patient with M0 CRPC and pelvic lymph nodes with less than 2.0 cm in minor axis do you recommend a different approach?							
	a) Apalutamide	b) Enzalutamide	c) Apalutamide or Enzalutamide	d) Bicalutamide	e) Docetaxel	f) Pelvic radiotherapy	g) Stereotactic body radiotherapy (SBRT) for oligometastatic lymph node	h) Lymphadenectomy
	1 (3.9%)	––	15 (54%)	1 (3.9%)	––	3 (11.5%)	6 (19.2%)	2 (7.7%)
13	When you recommend an antiandrogen to treat M0 CRPC, which one do you prefer?							
	a) Apalutamide	b) Enzalutamide	c) Apalutamide or Enzalutamide	d) Bicalutamide	e) Flutamide			
	6 (19.2%)	––	22 (80.8%)	––	––			
14	In a patient with no sign of metastatic disease based on regular exams (CT scan plus bone scan) and a positive PSMA-PET CT/MRI do you recommend a different approach?							
	a) Yes	b) No						
	13 (48%)	15 (52%)						
15	In a patient with no sign of metastatic disease based on regular exams (CT scan plus bone scan) and a positive PSMA-PET CT/MRI do you recommend?							
	a) Apalutamide	b) Enzalutamide	c) Apalutamide or Enzalutamide	d) Abiraterone	e) Bicalutamide	f) Docetaxel (based on volume of disease)	g) Local therapy if oligometastatic disease (SBRT, cryo, etc.)	h) It depends on PSA dT
	1 (3.9%)	1 (3.9%)	10 (38.5%)	2 (7.7%)	––	1 (3.9%)	7 (23.1%)	6 (19.2%)
16			Physicians: The drug preference is due to?					
	a) Toxicity profile	b) Experience	c) Cost	d) Access				
	22 (76%)	2 (8%)	––	4 (16%)				
17			When do you recommend stop treatment and start a new one?					
	a) PSA progression only	b) Clinical progression only	c) Radiological progression only	d) At least two of the three criteria (PSA, clinical and radiological)	e) Unequivocal clinical progression only	f) Unequivocal radiological progression only	g) Answers d, e, and f	h) All of the above
	––	––	––	15 (53.8%)	––	1 (3.8%)	12 (42.3%)	––
	What is your drug of choice for M0 CRPC in the majority of men with a reasonable life expectancy and…	a) Apalutamide or Enzalutamide	b) Apalutamide	c) Enzalutamide	d) Bicalutamide or Flutamide	e) Docetaxel	f) Abstain	
18	Diabetes?	27 (96.2%)	1 (3.8%)	––	––	––	––	
19	Mild hypertension?	26 (88.5%)	––	2 (7.7%)	––	––	––	
20	Moderate hypertension?	24 (84%)	4 (8.0%)	4 (8.0%)	––	––	––	
21	Severe hypertension?	20 (70.8%)	3 (12.5%)	5 (16.7%)	––	––	––	
22	Cardiovascular disease (cardiac insufficiency > grade 2)?	19 (66.7%)	7 (25.0%)	2 (8.3%)	––	––	––	
23	Cardiovascular disease (artery coronary disease)?	15 (51.9%)	9 (33.3%)	4 (14.8%)	––	––	––	
24	Past history of seizure and/ or drugs that increase risk of seizure?	4 (14.8%)	22 (77.8%)	––	2 (7.4%)	––	––	
25	Mental impairment disorder?	5 (19.2%)	23 (80.8%)	––	––	––	––	
26	Psychiatry disorders?	8 (28.0%)	20 (72.0%)	––	––	––	––	
27	Renal insufficiency?	24 (84.6%)	3 (11.5%)	1 (3.9%)	––	––	––	
28	COPD?	28 (100.0%)	––	––	––	––	––	
29	Past history of falls?	8 (29.6%)	11 (37.0%)	9 (33.3%)	––	––	––	
30	Multiple medications?	4 (14.3%)	3 (10.7%)	––	––	––	21 (75.0%)	
What is your level of concern with enzalutamide in the treatment of M0 CRPC regarding incidence of…		a) Low	b) Moderate	c) High	d) Abstain			
31	Fatigue?	2 (7.7%)	17 (61.5%)	9 (30.7%)	––			
32	Fracture?	16 (57.1%)	7 (25.0%)	5 (17.9%)	––			
33	Falls?	5 (18.5%)	18 (63.0%)	5 (18.5%)	––			
34	Hot flashes?	25 (82.1%)	2 (14.3%)	1 (3.6%)	––			
35	Nausea?	22 (78.6%)	6 (21.4%)	––	––			
36	Decreased appetite?	23 (82.1%)	5 (17.9%_	––	––			
37	Hypertension?	13 (42.9%)	15 (53.6%)	1 (3.6%)	––			
38	Cardiovascular complications?	14 (50.0%)	14 (50.0%)	––	––			
39	Mental impairment disorder?	7 (25.0%)	18 (64.3%)	3 (10.7%)	––			
40	Diarrhea?	26 (92.9%)	2 (7.1%)	––	––			
41	Rash?	26 (92.9%)	2 (7.1%)	––	––			
42	Arthralgia?	25 (89.3%)	3 (10.7%)	––	––			
43	Dizziness?	17 (60.7%)	11 (39.3%)	––	––			
44	Seizure?	14 (50.0%)	12 (42.9%)	2 (7.1%)	––			
45	Hepatic impairment?	28 (100.0%)	––	––	––			
What is your level of concern with apalutamide in the treatment of M0 CRPC regarding the incidence of…		a) Low	b) Moderate	c) High	d) Abstain			
46	Fatigue?	9 (30.8%)	16 (57.7%)	3 (11.5%)	––			
47	Fracture?	11 (39.3%)	13 (46.4%)	4 (14.3%)	––			
48	Falls?	10 (35.7%)	15 (53.6%)	3 (10.7%)	––			
49	Hot flashes?	13 (82.1%)	4 (14.3%)	1 (3.6%)	––			
50	Nausea?	22 (78.6%)	6 (21.4%)	––	––			
51	Decreased appetite?	24 (85.7%)	4 (14.3%)	––	––			
52	Hypertension?	10 (37.0%)	11 (40.7%)	7 (22.2%)	––			
53	Cardiovascular complications?	18 (64.3%)	10 (35.7%)	––	––			
54	Mental impairment disorder?	23 (82.1%)	5 (17.9%)	––	––			
55	Diarrhea?	23 (82.1%)	5 (17.9%)	––	––			
56	Rash?	8 (28.6%)	15 (53.6%)	5 (17.9%)	––			
57	Arthralgia?	24 (85.7%)	4 (14.3%)	––	––			
58	Dizziness?	24 (85.7%)	4 (14.3%)	––	––			
59	Seizure?	25 (89.3%)	3 (10.7%)	––	––			
60	Hepatic impairment?	27 (96.4%)	1 (3.6%)	––	––			
3. USE OF OSTEOCLAST-TARGETED THERAPY FOR SRE/SSE PREVENTION FOR ADVANCED PROSTATE CANCER (NOT FOR OSTEOPOROSIS/BONE LOSS)								
61	Which osteoclast-targeted therapy do you recommend for men with M0 CRPC for SRE/SSE prevention?							
	a) Zoledronic acid	b) Denosumab	c) Either zoledronic acid or denosumab	d) Another osteoclast- targeted therapy	e) I do not use osteoclast- targeted therapy in this setting, but may supplement calcium and vitamin D			
	1 (3.6%)	––	4 (14.3%)	––	23 (82.1%)			
When used for men with M0 CRPC, what treatment frequency do you recommend regarding…		a) Every 12 months	b) Every 6 months	c) Every 3 months	d) Every month	e) I do not use osteoclast- targeted therapy in this setting		
62	Zoledronic acid?	2 (8%)	2 (8%)	3 (12.0%)	––	21 (72.0%)		
63	Denosumab?	––	5 (19.3%)	1 (3.9%)	1 (3.9%)	21 (73.0%)		
74	For how long do you recommend osteoclast-targeted therapy for men with M0 CRPC for SRE/SSE prevention?							
	a) 1 year	b) 2 years	c) Until first SRE/SSE	d) Until second SRE/ SSE	e) Indefinitely	f) Until disease progression	g) I do not use osteoclast targeted therapy in this setting	
	––	2 (7.4%)	1 (3.7%)	1 ()3.7%	––	––	24 (85.2%)	

The SPARTAN trial showed rash as the most frequent AE in 23.8% of the patients receiving apalutamide and only 5.5% for the placebo arm. Of patients in the apatulamide arm, a grade 3 or 4 rash was present in 5.2% of them. Other frequent AEs in the apatulamide group versus the placebo group were fatigue (30.4% vs. 21.1%), hypertension (24.8% vs. 19.8%) and diarrhea (20.3% vs. 15.1%). Fractures occurred in 11.7% vs. 6.5% and hypothyroidism was found in 8.1% vs. 2%. Serious AEs were seen in 24.8% (199) of the apatulamide arm and 23.1% (92) of the patients in the placebo arm. Discontinuation due to AE was 10.6% (85 patients) in the apatulamide arm and 7% (28 patients) in the placebo group.

The PROSPER trial had 10% of patients discontinue enzalutamide due to AEs compared to 8% of placebo patients. The only AE that occurred in more than 20% of the enzalutamide patients was fatigue with 33% vs. 14% in the placebo arm. Falls and non-pathologic fractures occurred in 17% of the enzalutamide group vs. 8% for placebo. Hypertension was reported in 12% vs. 5%, major cardiovascular events in 5% vs. 3%, and mental impair ment disorders in 5% vs. 2%. These data show that it is crucial to weigh advantages against potential harms.

Bicalutamide was included as a survey answer for treatment alternatives. This particular drug has two common AEs (breast pain and gynecomastia), which can reduce QOL. Throughout trials, several patients dropped out of the study due to these AEs (20). Another treatment alternative option for this survey was flutamide. This treatment has two common AEs (gynecomastia and gastrointestinal upset) as well as some other AEs, such as severe liver toxicity and hot flashes (21).

Docetaxel was also provided as a survey option for treatment. AEs of this drug are usually due to dosing and include neutropenia, hypersensitivity, fluid retention, nail toxicities, neuropathy and asthenia. Of note, toxicities are the main concern of this medication, given that there is insufficient guidance on how to manage this side effect. Myelosuppression is the most frequent toxicity associated with docetaxel dosing (22). Currently, there is no randomized trial evaluating docetaxel in M0 CRPC.

Additionally, abiraterone acetate was included as an alternative survey answer. The most common AEs of abiraterone are grade 1 and 2. These AEs include toxicities, fatigue, hypertension, headache, nausea, and diarrhea. Grade 3 AEs of abiraterone are hypertension, hypokalemia, constipation, diarrhea, muscular weakness, and arthralgia (23). Finally, radium-223 is generally used when PCa has metastasized to bone. AEs of radium-223 include nausea, fatigue, vomiting, diarrhea, constipation, bone pain, urinary tract infection and peripheral edema. Hematological AEs of radium-223 are generally mild (24). There are no randomized controlled trials completed for abiraterone or radium-223 in M0 CRPC.

The panel did not reach consensus regarding their level of concern with either apalutamide or enzalutamide regarding fatigue, fractures, or falls. For apatulamide, the majority of the panel has a moderate concern related to fatigue (58%), fractures (46%) and falls (53%). For enzalutamide, the majority of the panel has low concern regarding fractures (57%) but kept moderate concern for fatigue (62%) and falls (63%). The highest percentage of concern was a moderate level of concern regarding the incidence of falls when treating M0 CRPC patients with enzalutamide (63%). The second highest percentage was 62%, which correlated with a moderate level of concern for fatigue as an AE when using enzalutamide in the treatment of M0 CRPC patients. All other answers had various votes and percentages ranging from low, moderate, and high levels of concern for those specific AEs.

Contrastingly, all answers reached consensus stating physicians have a low level of concern for AEs such as hot flashes (82% and 82%), nausea (78% and 78%) and decreased appetite (85% and 82%) with either apalutamide or enzalutamide respectively. All answers related to these AEs had less than 5% of voters stating a high level of concern with either of these side effects when treating their M0 CRPC patients with these new hormonal agents. Additionally, for other AEs, such as hepatic impairment (96% and 100%), diarrhea (82% and 93%) and arthralgia (86% and 89%), the panel reached consensus on a low level of concern when treating patients with either apalutamide or enzalutamide respectively.

Cardiovascular AEs, such as hypertension or other cardiovascular complications, are not a major concern for the panel when treating patients with apalutamide or enzalutamide. The majority of the panel members voted low or moderate level of concern for these side effects. For apalutamide and hypertension, 41% have moderate concern, 37% have low concern, and 22% voted high level of concern. For enzalutamide and hypertension, 54% voted for moderate concern, 43% low concern, and 4% chose high level of concern. Regarding the panel's concern for “other cardiovascular complications”, all votes were within the low and moderate range. For apalutamide, 64% voted for low level and 36% for moderate concern. For enzalutamide, the panel was divided in half between low and moderate level of concern with 50% each.

Mental disorders as an AE were also of low and moderate concern for the treating physicians in the panel. However, consensus was only reached about apalutamide, given the majority of the attendees have a low level of concern regarding mental disorders (82%). For enzalutamide, 63% of the panel voted for moderate level of concern and 25% for low level of concern. Other central nervous system AEs, such as dizziness and seizures, are a low level of concern to the panel when using apalutamide (85% for dizziness and 90% for seizures). However, when using enzalutamide, the panel did not reach consensus and a higher percentage of voters expressed a moderate level of concern for both dizziness and seizures in their patients (39% and 43%).

Rash has been seen as an AE in the international trials for apalutamide. The majority (53%) of the panel have moderate concern when treating M0 CRPC with apalutamide, 29% have low concern, and 18% have high concern. Contrastingly, for enzalutamide, 93% of the panel voted for low concern and 7% for moderate concern.

Overall, when asked about levels of concern regarding the incidence of AEs with apalutamide and enzalutamide for M0 CRPC patients, treating physicians showed low or moderate levels of concern for most AE. These data indicate a support for the wide use of these drugs. There were only two instances where more than 20% of the panel voted with high level of concern: fatigue with enzalutamide (33%) and hypertension with apalutamide (22%). Again, these results are unique due to the fact that no guidelines have fully explored the toxicity profile of the active agents and how this may influence a physician's drug choice (16, 19).

## USE OF OSTEOCLAST-TARGETED THERAPY FOR THE PREVENTION OF SKELETAL-RELATED EVENTS AND SYMPTOMATIC SKELETAL EVENTS IN M0CRPC (NOT FOR OSTEOPOROSIS/BONE LOSS)

Bone is the most common metastatic site and a major cause of morbidity for patients with PCa, however, the use of bone-targeted therapies such as zoledronic acid and denosumab (RANK-L monoclonal antibody) have reduced time to first SREs/SSEs (25–27).

Denosumab has been evaluated in a randomized trial that included 1432 patients with M0 CRPC. In this trial, denosumab significantly increased bone-MFS by a median of 4.2 months compared with placebo (hazard ratio [HR] 0.85, 95% CI 0.73-0.98, p=0.028). Despite these results, denosumab has not been approved for this patient population, as this was not considered clinically significant (7).

Per the consensus, 82% of the panel does not recommend the use of any osteoclast targeted therapy for SRE/SSE prevention in M0 CRPC patients, giving indication for the response stratification in the questions related to the treatment frequency and length. Regarding zoledronic acid or denosumab treatment frequency, the majority of the voters selected they do not use osteoclast––targeted therapy in these patients (~70% for both). Consensus was emphasized when asked about the length of these treatments for M0 CRPC patients, where 85% of the panel responded that osteoclast-targeted therapy is not used in this setting. These results are in accordance with the NCCN guidelines version 1 2020 (17), which does not support the use of osteoclast-targeted therapy in these patients.

## CONCLUSIONS

M0 CRPC affects a significant proportion of PCa patients who failed local therapy (either surgery, radiation, or both). M0 CRPC is associated with an elevated probability of bone metastases development as well as SREs, decreased QOL and survival, particularly in those with accelerated PSAdt. Until recently, there were no systemic agents that significantly changed the natural history of this disease. Three randomized trials, PROSPER (enzalutamide), SPARTAN (apalutamide) and ARAMIS (darulotamide), have shown benefit in MFS and other secondary clinically relevant endpoints in a well-defined patient population. Although these drugs were tested in large phase III trials compared to placebo, none of them were compared to each other in a M0 CRPC setting. In addition, these drugs have not yet been tested in other subgroups of patients (e.g., PSAtd ³10 months) and are associated with different toxicity profiles.

This survey has potential limitations. The expert's opinions may not reflect the reality regarding access to newer diagnostic tools or treatment modalities, especially in the public health system. Economic disparities among different regions in Brazil may be a potential bias in the choice of treatments. Moreover, the absence of consensus on some topics might reflect lack of high quality evidence at this point. Notably, this consensus recognized the different toxicity profiles of apalutamide and enzalutamide that may have clinical implications on the choice of treatment according to specific patient's comorbidities. Finally, some expert's insights have been provided on the use of apalutamide and enzalutamide in patients with M0 CRPC who were not eligible for PROSPER or SPARTAN due to clinical and laboratory factors, such as shorter life expectancy and longer PSAtd, with the goal to facilitate clinical decision making..
